# Aspirin for the prevention of migraine in pregnancy: A retrospective, descriptive case series from a tertiary centre

**DOI:** 10.1016/j.clinme.2026.100648

**Published:** 2026-09-24

**Authors:** Cleodie Swire, Melanie Nana, Catherine Tew, Catherine Williamson, Catherine Nelson-Piercy

**Affiliations:** a Department of Clinical Pharmacology and Therapeutics, St George’s Hospitals NHS Foundation Trust, London, United Kingdom; b Department of Obstetric Medicine, Guy’s and St Thomas’ Hospitals NHS Foundation Trust, London, United Kingdom; c Department of Obstetrics and Gynaecology, Guy’s and St Thomas’ Hospitals NHS Foundation Trust, London, United Kingdom; d Department of Metabolism, Digestion and Reproduction, Imperial College London, United Kingdom

**Keywords:** Migraine, Pregnancy, Aspirin

## Abstract

**Study objective:**

To assess the use of low-dose aspirin for the prevention of migraine in pregnant women.

**Design:**

This was a single-centre, retrospective, descriptive case series. Data were obtained through a retrospective review of electronic clinical records. Data are reported on migraine history and pharmacological management of migraine prior to and during the index pregnancy, as well as maternal and neonatal outcomes.

**Setting:**

A specialist obstetric medicine clinic in London, UK.

**Participants:**

Pregnant women seen due to headache in the clinic between January 2020 and August 2023, with a diagnosis of ‘migraine’ in their electronic clinical records were selected for this study. 73 patients were eligible for inclusion.

**Interventions:**

Patients received routine clinical care as determined by the clinical team at the time; no study-specific interventions were applied.

**Main outcome measure(s):**

The main outcomes were frequency of low-dose aspirin use for migraine prevention in pregnancy, and change in migraine symptoms after aspirin was started for migraine prevention.

**Results:**

Pharmacological prevention for migraine was used by 66/73 (90%) women. Low-dose aspirin was taken for migraine prevention by 61/73 (86%); 36/61 (59%) had aspirin monotherapy and the remainder took more than one medication for this indication. In the aspirin monotherapy group, 30/31 (97%, 5/36 not documented) subsequently reported an improvement in their migraine. Maternal and neonatal outcomes were consistent with baseline data for the population.

**Conclusion:**

This case series adds to the literature describing use of low-dose aspirin for migraine prevention in pregnancy.

## Highlights

• Migraine often, but not universally, improves in pregnancy. In some cases, pharmacological prevention is required.
• Low-dose aspirin is safe in pregnancy. There is evidence of efficacy in preventing migraine in some populations.
• This retrospective case series describes 73 pregnant women living with migraine. 61 took aspirin to prevent migraine symptoms.
• Of 31 women taking aspirin who had follow-up where migraine severity was re-assessed at follow-up; 30/31 (97%) reported migraine improvement.

## Graphical Abstract

**Figure:**
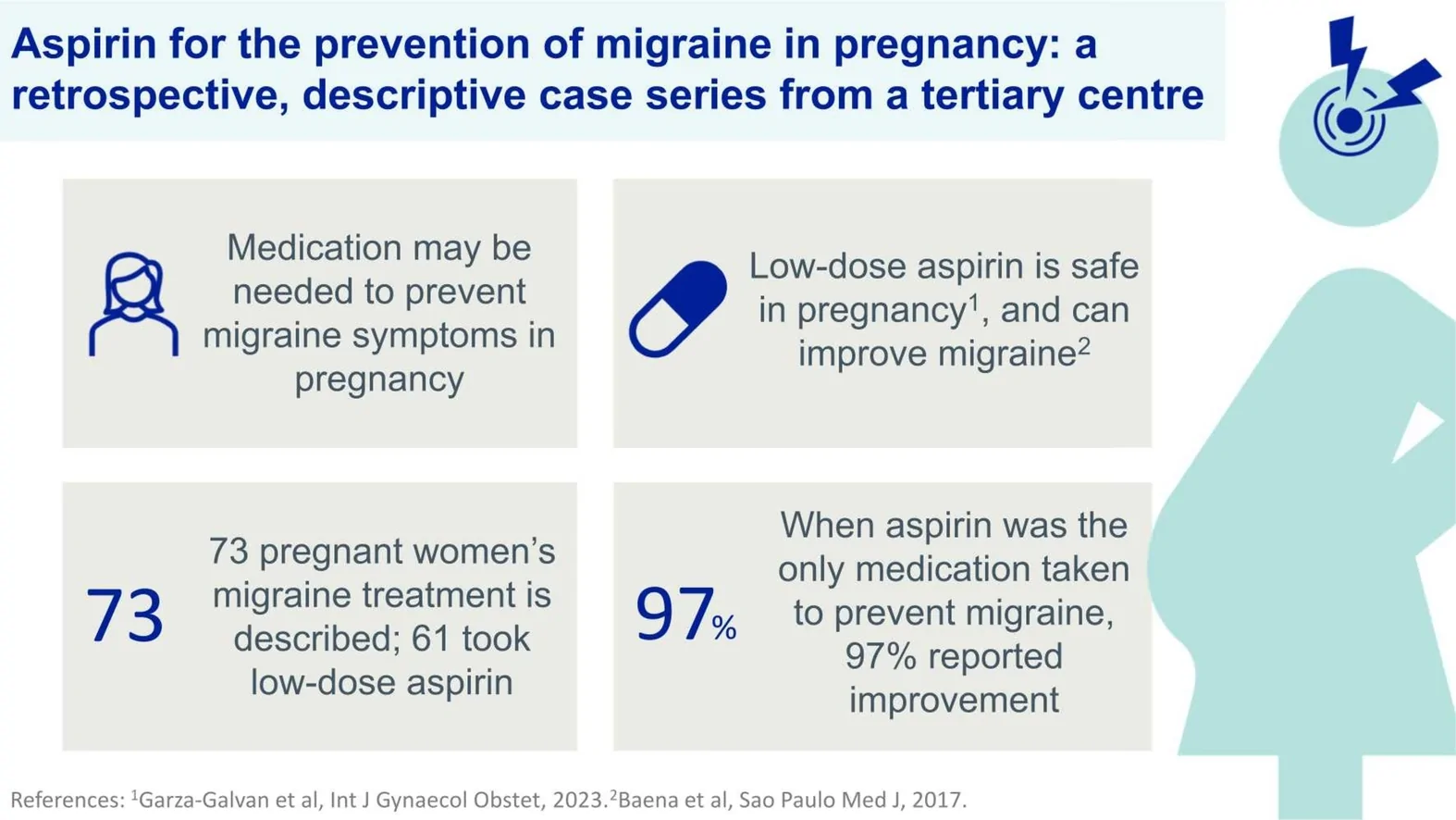

## Introduction

Migraine is common in women of childbearing age,^1^ representing the leading cause of years lived with disability in this age group.^2^ Migraine is therefore commonly encountered in pregnancy, representing the most common headache disorder for which pregnant women seek medical attention.^3^

Migraine in pregnancy is associated with increased risk of pregnancy-induced hypertension and pre-eclampsia.^4^, ^5^ An association has also been found between intra/peri-partum strokes and migraine, which persists once adjustments are made for hypertension within the study population.^6^ Additionally, the condition has a significant impact on an individual’s wellbeing and ability to function in their personal and/or professional life.^7^

In a review of observational studies, it was reported that 60–70% of women living with migraine experience improvements in symptoms during pregnancy.^8^ However, this is not the case for all, and remission of migraine is less common in those with a history of aura.^9^ Migraine may also present *de novo* in pregnancy; in a large prospective study this was found to be the case in 14% of cases.^10^

Outside of pregnancy, the National Institute for Health and Care Excellence (NICE) guidance recommends preventive treatment for migraine if: they have a significant impact on quality of life, they occur frequently, they are prolonged/severe despite optimal acute treatment, or the person is at risk of medication overuse headache.^11^ Preventive therapies endorsed by NICE include propranolol, amitriptyline and topiramate, the latter of which is teratogenic and contraindicated in pregnancy. Evidence for low-dose aspirin to prevent migraine is of poor quality and largely reported in male subjects.^12^, ^13^, ^14^ Published literature describing the use of low-dose aspirin for the prevention of migraine in pregnancy is limited, to the best of our knowledge, to one letter published in 1996 that reported subjective improvement in symptoms in 22 out of 28 (78.6%) women using aspirin for migraine prevention in pregnancy.^15^ We propose that low-dose aspirin, for which extensive safety data in pregnancy exist,^16^ may provide an additional valuable choice as a preventive in this population. This study describes use of low-dose aspirin for the prevention of migraine in pregnant women in our centre, reporting maternal and neonatal outcomes.

## Material and methods

### Study design

This study was a single-centre, retrospective, descriptive case series.

### Setting

The setting for this study was Guy’s and St Thomas’ Hospital NHS Trust (GSTT) in London, UK. A retrospective analysis of patients seen between January 2020 and August 2023 was carried out between August 2023 and August 2024. This study was deemed exempt from requiring ethical approval and individual patient consent because it is a retrospective evaluation of patients identified in an anonymised database. The Health Research Authority decision tool was used; the outcome was that this study would not be considered research by the NHS.

### Participants

All cases of pregnant women coded as being consulted for ‘migraine’ on the obstetric medicine clinic’s database in the study period were selected for this study.

Women were excluded if they were not pregnant at the time of the appointment. Some women gave birth at another hospital and were excluded from the analysis as outcome data were not available. Migraine cases were defined as where ‘migraine’ was the reason for the consultation documented in the clinical notes. Due to the retrospective nature of this study, it was not possible to determine the diagnostic process for migraine for individual women prior to or at their obstetric medicine clinic appointment.

### Variables

Data were collected on women’s demographic details, previous obstetric history, features of and management of migraine historically and during the index pregnancy in addition to maternal and neonatal outcomes.

Improvement in migraine severity was defined by: documentation of self-reported subjective improvement in migraine severity in the electronic patient records at a subsequent obstetric medicine or other antenatal clinic appointment. Improvement could be either reduced frequency or reduced severity of migraine symptoms, or both.

### Data sources/measurement

Data were abstracted by one reviewer from the electronic clinical notes of women who met the selection and eligibility criteria. Data were collected and stored in an anonymised spreadsheet.

### Bias

Where data for variables were missing for individual women, this information has been reported to aid interpretation. As women with more complex or resistant medical problems are referred to obstetric medicine clinics, this population is not likely to be representative of all women living with migraine in pregnancy.

### Statistical analysis

Characteristics were summarised using descriptive statistics. Counts and percentages are provided for categorical variables. The reported continuous variables were assessed for distribution using visual and numerical methods in Microsoft Excel^17^ to determine which descriptive statistics to use. For the primary outcome (improvement in migraine symptoms for women who received aspirin monotherapy to prevent migraine), Clopper–Pearson exact confidence interval was calculated using R.^18^

## Results

During the three year and eight-month study period 2,693 new patients were reviewed in the obstetric medicine clinics of which 89 (3%) were coded in the clinic’s database as being seen due to migraine. In total, 16 were excluded based on the above criteria (see Fig. 1). A total of 73 patients is therefore included in the report.

**Fig. 1:**
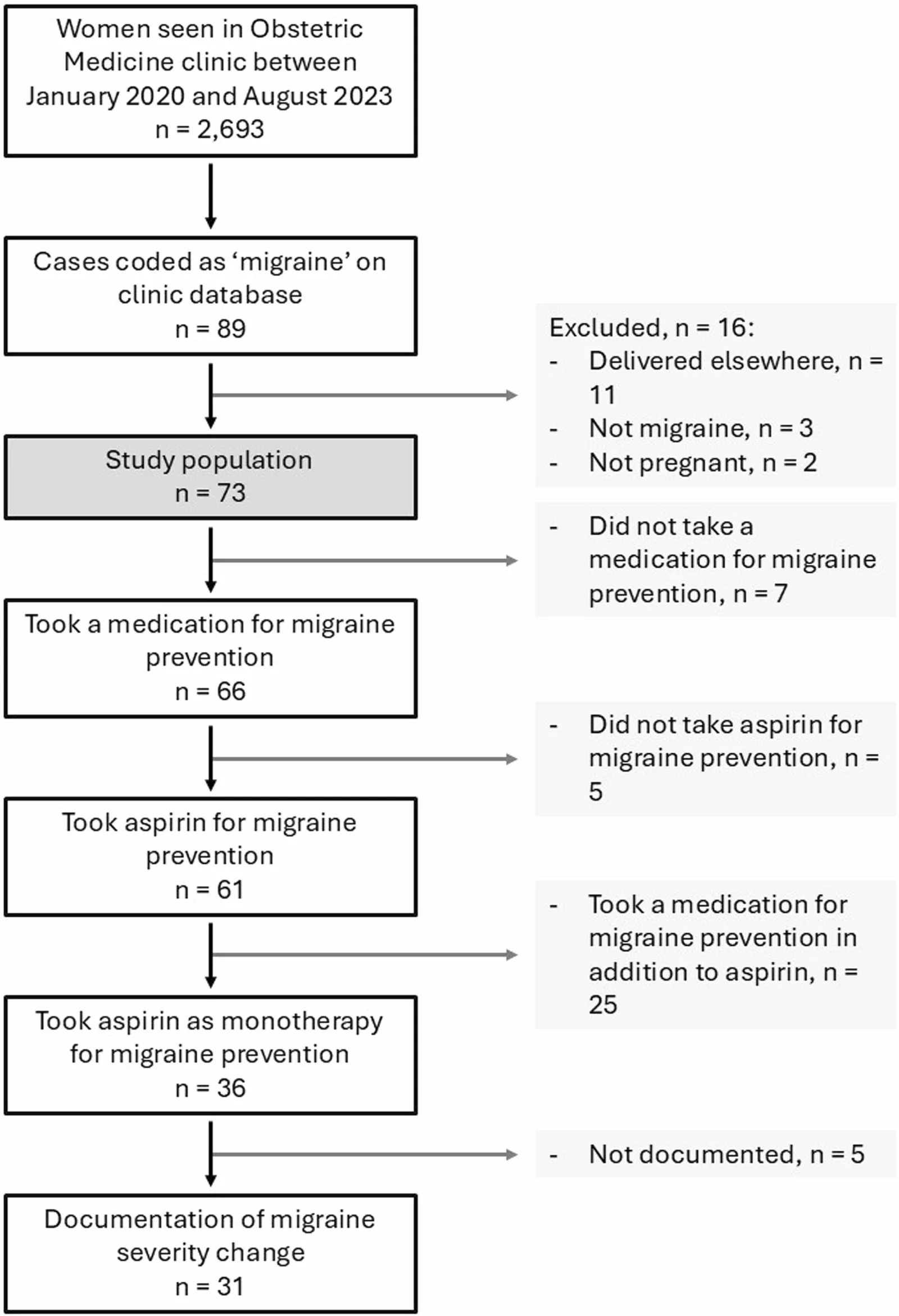
Flow diagram identifying the study population from all clinic attendees, showing sequential exclusions and final analytic groups, with number (n) of eligible and excluded women at each step.

On assessment of distribution for continuous variables, numerical skewness values were modest for two variables (−0.4 for age at first appointment and 0.5 for BMI at booking) and suggestive of positive skew for a third (1.0 for gestation at first appointment). All three variables showed asymmetry on visual inspection; therefore, median values and interquartile ranges are provided for continuous variables.

### Maternal demographics

The demographics and disease characteristics of the women are described in Table 1. Throughout the report the denominator represents the total number of patients in which each of the outcomes reported were available; data were not available for the whole cohort for certain outcomes. In total, 65/73 (89%) had a pre-existing diagnosis of migraine prior to pregnancy. Of these, 23/30 (77%, 35/65 not documented) women’s historical migraine occurred with a frequency of at least once a month.

**Table 1:** Maternal demographics and history of migraine prior to the index pregnancy.

**Maternal demographics**	
Maternal age at first appointment (years), median (IQR) (n = 73)	33.8 (29.8–36.0)
BMI at pregnancy booking (kg/m^2^), median (IQR) (n = 73)	23.8 (20.9–28.6)
Ethnicity, n (%) (n = 73) − White − Asian − Black − Mixed − Other	41 (56%) 8 (11%) 13 (18%) 8 (11%) 3 (4%)
Parity, n (%) (n = 73) − 0 − 1 − 2 − 3 − 4	39 (53%) 21 (29%) 9 (12%) 2 (3%) 2 (3%)
**Migraine history**	
− History of migraine prior to this pregnancy, n (%). (n = 73) − Yes − No	65 (89%) 8 (11%)
Historical migraine frequency, n (%). (n = 30; not documented in 35 cases) − Less than once per month − 1–4 days per month − 5–29 days per month − Daily	7 (23%) 15 (50%) 6 (20%) 2 (7%)
History of headache with migraine, n (%). (n = 61; not documented in four cases) − Yes − No	61 (100%) 0 (0%)
History of aura with migraine, n (%). (n = 45; not documented in 20 cases) − Yes − No	23 (51%) 22 (49%)
History of menstruation-related migraine, n (%). (n = 9; not documented in 56 cases) − Yes − No	6 (67%) 3 (33%)

IQR, inter-quartile range; n, number of cases.

### Historical management of migraine

In total, 15/64 (23%, 1/65 not documented) women reported previously having taken medications for migraine prevention, and five of these had taken more than one medication type. Where previous medications used were documented (57/65 cases), these included beta blockers (n = 8), amitriptyline (n = 7), aspirin (n = 2), topiramate (n = 2) and betahistine (n = 1).

In terms of acute treatment, 43/49 (88%, 16/65 not documented) women reported that they had previously taken medication for migraine, and 20 of these had used more than one medication type. Medications used were paracetamol (n = 25), non-steroidal anti-inflammatory drugs (NSAIDs) (n = 14), triptans (n = 14), weak opioids (n = 9), aspirin (n = 2), metoclopramide (n = 1) and gabapentin (n = 1).

### Headache characteristics and management in pregnancy

Of the women who had migraine in pregnancy, 66/68 (97%, 5/73 not documented) experienced headache and 24/51 (47%, 22/73 not documented) had aura (two of whom did not have co-existent headache). The median gestation at first appointment was 17 + 1 (13 + 6–21 + 5, n = 73) weeks’ gestation. At their first appointment, 26/33 (79%, 40/73 not documented), reported that their migraine frequency had increased, seven reported no change and none reported a decrease.

Overall, 66/73 (90%) women took medications for migraine prevention during their pregnancy; 25 took more than one medication. In total, 61/73 (84%) took aspirin, 19 amitriptyline, 15 a beta blocker, three had a greater occipital nerve block and one used betahistine.

Of patients with pre-existing migraine 50/57 (88%, 8/65 historic medications not documented) had an alteration in migraine preventive compared to outside of pregnancy. Of the total cohort, 51/65 (78%, 8/73 historic medications not documented) took a preventive medication for the first time. In total, 11/15 (73%) who were taking preventive medications outside of pregnancy continued to take them from the start of their pregnancy. These medications included amitriptyline or a beta blocker as monotherapy or in combination, and in one case betahistine monotherapy. One woman was advised to restart taking her usual preventive medication of amitriptyline and a beta blocker as the first intervention.

Of the cohort who took aspirin, 57/60 (95%, 1/61 not documented) took 75 mg and three women took 150 mg. Of the aspirin sub-group 11/61 (18%) women had been advised during the index pregnancy to start aspirin for another indication prior to their first appointment for migraine; ten for pre-eclampsia prevention and one due to acute presentation to hospital with migraine. 41/61 (67%) started aspirin at their initial obstetric medicine clinic appointment, and 9/61 (15%) after their appointment. All these women started aspirin for the indication of migraine prevention. Of the women who took aspirin, 36/61 (59%) took this as monotherapy for migraine prevention. 30/31 (97%, 5/36 not documented; (95% Clopper–Pearson exact confidence interval: 0.83–1.00) of this sub-group had an improvement in their migraine. Of women who took aspirin as monotherapy for migraine prevention who also had a history of aura, 12/13 (92%) reported a subjective improvement in their migraine symptoms. Of the full aspirin cohort (including those taking other migraine preventives), 52/58 (90%, 3/61 not documented) reported subjective improvement in migraine symptoms.

For acute management of their migraine, 65/71 (92%, 2/73 not documented) women took medications, 46 of whom took more than one medication. Of these, 61 used paracetamol, 30 an NSAID, 18 a triptan, 18 a weak opioid, two took metoclopramide and one gabapentin.

### Pregnancy outcomes

There were no miscarriages in this cohort; one woman elected to have a termination of pregnancy (reason unknown) and there were 72 live births. In terms of maternal outcomes, 3/72 (4%) women developed pregnancy-induced hypertension and 2/72 (3%) developed pre-eclampsia.

### Obstetric and neonatal outcomes for women who took aspirin

Obstetric outcomes for the sub-group who took aspirin (n = 61) are described in Table 2. There were 60 live births, no stillbirths and one termination of pregnancy. Median gestation at birth was 39 + 1 weeks’. In total, 53% were born by caesarean section. Overall, 7/28 (25%) women with a vaginal delivery had an estimated blood loss in excess of 500 mL, and 6/32 (19%) women with a caesarean birth had blood loss of 1 L or more.

**Table 2:** Obstetric and neonatal outcomes for women who took aspirin.^a^

All births, n	60
Multiple births, n. n = 60	0
Gestation at birth (weeks + days), median (IQR). n = 60	39 + 1 (38 + 1–40 + 1)
Pre-term birth, n (%). n = 60	5 (8%)
Mode of delivery, n (%). n = 60	
− Spontaneous vaginal birth − Assisted vaginal birth − Elective caesarean section − Emergency caesarean section o Category 1 o Category 2 o Category 3	25 (42%) 3 (5%) 15 (25%) 17 (28%) 3 (5%) 9 (15%) 5 (8%)
Maternal bleeding complications, n (%)	
− Vaginal birth, estimated blood loss. n = 28 o 0.5–1 L o 1–1.5 L o 1.5–2 L o 2–2.5 L − Caesarean birth, estimated blood loss. n = 32 o 0.5–1–L o 1–1.5 L o 1.5–2 L o 2–2.5 L − Other	6 (21%) 1 (4%) 0 0 18 (56%) 5 (16%) 0 1 (3%) 0
Neonatal bleeding complications, n (%). n = 60	0
Congenital abnormalities, n (%). n = 60	2 (3%)

IQR, inter-quartile range; n, number of cases.

a Case where pregnancy outcome was termination of pregnancy excluded.

There were no neonatal bleeding complications, and 2/60 (3%) neonates had congenital anomalies. In one case the anomaly was a tongue tie, and in one case the neonate had pseudosquint, a small skin cyst and superficial capillary-like vascular malformations over the occiput and neck.

## Discussion

### Key results

This 44-month study provides retrospective data on 73 women living with migraine in pregnancy, 61 of whom took low-dose aspirin for migraine prevention. Key findings include that 97% of women taking aspirin as monotherapy for prevention reported improvements in migraine. This must be interpreted with caution; it is known that many women experience improvement in migraine in pregnancy, and it is not possible to distinguish whether this result relates to a therapeutic effect of aspirin or natural pregnancy-associated improvement. When compared to the background pregnant population there was no increase in severe bleeding complications.

In our obstetric medicine clinics 3% of patients had a consultation due to migraine. This is in keeping with migraine being a common condition in women of child-bearing age, with mean global prevalence in females aged 20–64 estimated as 19%.^19^ In this study, 11% of cases seen in the clinics were related to a new diagnosis of migraine in pregnancy, which is in keeping with a rate of 14% in a previous study.^10^

The number of women reporting a deterioration in their migraine at their first appointment was 79%; it is not possible to compare this to the proportion of women who have deterioration in migraine in the background pregnant population as the clinic only sees women who have symptoms in pregnancy. In total, 77% of the women in this study had a pre-pregnancy migraine frequency of at least once a month, which compares to 62% in a large USA study.^20^ The incidence of aura in our study is higher than background rates, with 51% reporting aura compared to around 30% in broader migraine populations.^21^ Some headache specialists have suggested that aspirin may have particular benefits for patients with aura,^22^ although this has not been compared through direct comparison with appropriate adjustment for confounders.

This study confirms that low-dose aspirin is the most commonly prescribed migraine preventive in our service (used in 84% cases). Of those who were prescribed aspirin as monotherapy, 97% had an improvement in their migraine. Systematic review of published trials on aspirin use for migraine prevention outside of pregnancy is limited by various factors including variation in aspirin dose, changes in diagnostic criteria for migraine over time and methodology for determining improvements. However, a review of eight papers including 28,326 participants (96% of whom were male) concluded that six of these studies showed that migraine frequency reduced if aspirin dose was at least 325 mg per day.^12^ This is a higher dose than was routinely prescribed in our clinic. The pre-existing evidence for aspirin use in migraine prevention is primarily based on two large-scale trials involving male doctors in the UK and USA in the 1980s which found that individuals in the study group who had a history of migraine reported improvements in their headaches.^13^, ^14^ Both these trials were investigating aspirin for primary prevention of cardiovascular disease and carried out a secondary analysis on migraine. Both trials had limitations relating to their design. For example, there was no randomisation between the study and control groups based on existing migraine diagnosis, symptoms were self-reported and not standardised, and there was no placebo group in the Peto *et al*. study. In women, a study evaluating migraine in 1,001 middle-aged females found trends towards improvements in migraine severity and duration in a group taking 100 mg aspirin every other day compared to placebo, although these differences were not statistically significant.^23^

### Limitations

Data quality and completeness were limited by the retrospective nature of the study. Our data were extracted from contemporaneous free-text documentation which resulted in incomplete data, inconsistent data entry and the potential for errors. Data standardisation was also challenging as assessment of symptoms was based on subjective statements and did not use a verified grading system for migraine. Nevertheless, this is the largest reported cohort of pregnant women receiving low-dose aspirin for migraine prevention, and we have been able to report both maternal and neonatal outcomes for most patients.

Case selection was dependent on coding within a database. This is a potential source of bias as it is possible some cases were not identified. In addition, the study population was limited to women living with migraine referred to the obstetric medicine service and did not include those whose migraine symptoms improved in pregnancy or those not referred. The higher rates of aura in this cohort compared to the baseline rate may reflect a bias towards people with aura being more likely to be referred for specialist input.

Without a comparator group (either women who used another preventive medication, or who did not take a migraine preventive in pregnancy), definitive conclusions about whether the improvement rate is due to the effect of aspirin or natural time-dependent change cannot be made.

### Interpretation

In the context of pregnancy, our data add to a letter reporting outcomes of a group of 37 women with frequent or severe migraine symptoms in pregnancy.^15^ In that cohort 28 (76%) of the women took 75 mg aspirin for migraine prevention of whom 22 (79%) reported subjective improvement in symptoms. In our study, the proportion who had subjective improvements in migraine symptoms was also high at 97%. We acknowledge, however, that the natural history of migraine in pregnancy is such that we would anticipate that it would improve with increasing gestation for many women. A prospective study found that 50–80% of women living with migraine experience an improvement in the frequency and severity of migraine episodes during pregnancy, especially after the first trimester.^24^ Our study, which relates to the care of women with frequent and/or severe migraine in pregnancy, adds to the previously published letter in describing the use of low-dose aspirin for migraine prevention in pregnancy.

Extensive evidence supports the safety of low-dose aspirin in pregnancy.^16^ Maternal and neonatal outcomes from our study were in keeping with local and national statistics. However, our cohort are not representative of the general pregnant population so comparative rates should be interpreted with caution due to the risk of confounding. In our aspirin sub-group, rates of mode of delivery were 42% spontaneous vaginal birth, 5% assisted vaginal birth, 25% elective caesarean section and 28% emergency caesarean section; in 2022–2023 the data for GSTT were 40%, 11%, 20% and 29% respectively.^25^ The pre-term birth rate was 8% in our study; the rate for England and Wales in 2022 was 7.9%.^26^ One woman (2%) had PPH of 1.5 L or more in our cohort; GSTT data from January to March 2022 had a rate of 4%.^27^ The 3% rate of congenital anomaly in this study compares to a rate of 2.35% in England in 2021.^28^

Women living with migraine have been found to have higher rates of cardiovascular disease in pregnancy including hypertension,^5^ pre-eclampsia^4^ and strokes.^6^ Overlapping pathophysiological features include inflammation, platelet activation and vascular endothelial dysfunction. Aspirin has been demonstrated to inhibit such inflammation and platelet aggregation and thus reduce the risk of pre-term pre-eclampsia.^29^, ^30^ Absolute numbers were small but the proportion of the women in our cohort who developed pregnancy-induced hypertension and pre-eclampsia were 4% and 3%, respectively. An analysis of national data showed 2% women had pre-eclampsia in pregnancy,^31^ and around 10% of women have hypertension in pregnancy. The incidence of each condition in our cohort was no higher than the general population, although valid conclusions cannot be drawn due to differences between our cohort and the general population as well as the limited cohort in our study. Future studies should include the role of low-dose aspirin as a migraine preventive in pregnancy and possible benefits in reducing the risk of hypertensive complications in this population.

### Generalisability

The cohort described in this study were seen at a specialist physician-led obstetric medicine clinic at a tertiary hospital, and the findings may not be generalisable to other hospitals. Women seen in this clinic had migraine symptoms in pregnancy; those living with migraine who are asymptomatic in pregnancy are not reflected in this cohort.

## Conclusions

In this single centre study, we have described a cohort of 73 women who had symptomatic migraine during pregnancy. 84% of the women were prescribed low-dose aspirin as a migraine preventive. 97% of women who took aspirin as monotherapy reported improvement in their migraine symptoms, but causality for this improvement should not be directly inferred. This adds to the evidence reporting the use of low-dose aspirin for migraine prevention in pregnancy. There is evidence that migraine is associated with cardiovascular disease during pregnancy, and future work can consider whether there is a role for aspirin in reducing this risk.

## CRediT authorship contribution statement

**Cleodie Swire:** Writing – review & editing, Writing – original draft, Visualization, Investigation, Formal analysis. **Melanie Nana:** Writing – review & editing, Writing – original draft, Visualization, Methodology, Conceptualization. **Catherine Tew:** Writing – review & editing, Investigation. **Catherine Williamson:** Writing – review & editing. **Catherine Nelson-Piercy:** Writing – review & editing, Supervision, Conceptualization.

## Ethics approval and consent to participate

This study was deemed exempt from requiring ethical approval and individual patient consent because it is a retrospective evaluation of patients identified in an anonymised database. The Health Research Authority decision tool was used; the outcome was that this study would not be considered research by the NHS.

## Funding

Melanie Nana is funded by an 10.13039/100006662NIHR Clinical Research Training Fellowship. Catherine Williamson is funded as a principal applicant by Tommy’s Centre Grant Award, Mirum Pharmaceuticals Investigator Led Grant and 10.13039/501100000265MRC CASE PhD studentship.

## Declaration of competing interest

The authors declare that they have no known competing financial interests or personal relationships that could have appeared to influence the work reported in this paper.

## Data Availability

Data are available from the authors upon reasonable request.
